# Syntheses and antibacterial activities of 4 linear nonphenolic diarylheptanoids

**DOI:** 10.3906/kim-1911-61

**Published:** 2020-06-01

**Authors:** Şemsi Betül DEMİR, Hatice SEÇİNTİ, Neslihan ÇELEBİOĞLU, Murat ÖZDAL, Alev SEZEN, Özlem GÜLMEZ, Ömer Faruk ALGUR, Hasan SEÇEN

**Affiliations:** 1 Department of Chemistry, Faculty of Sciences, Atatürk University, Erzurum Turkey; 2 Department of Biology, Faculty of Sciences, Atatürk University, Erzurum Turkey

**Keywords:** Linear diarylheptanoid, curcuminoid, alnustone, natural product, synthesis, antimicrobial activity

## Abstract

Four linear nonphenolic diarylheptanoids were synthesized and their antibacterial activities were studied. ( *S* )-2-Me-CBS-catalysed reduction of alnustone with BH_3_SMe_2_ gave ( *R* )(-)(4 *E* ,6 *E* )-1,7-diphenylhepta-4,6-dien-3-ol, a natural product. Reduction of alnustone with Na in *t* -BuOH at –15 °C under N_3_ atm gave (_E_)-1,7-diphenylhept-5- en-3-one as a Birch-type reduction product. _t_-BuOK catalysed condensation of benzalacetone with propionyl chloride gave (4 *Z* ,6 *E* )-5-hydroxy-1,7-diphenylhepta-4,6-dien-3-one, a natural product. (1 *E* ,4 *Z* ,6 *E* )-5-Hydroxy-4-phenethyl-1,7-diphenylhepta-1,4,6-trien-3-one, a curcuminoid, was synthesized starting from pentan-2,4-dione in 3 steps. The synthesized chemical compounds were applied against 2 gram-positive bacteria ( **Bacillus cereus** and **Arthrobacter agilis** ), 4 gram-negative bacteria ( **Pseudomonas aeruginosa** , **Xanthomonas campestris** , **Klebsiella oxytoca** , and **Helicobacter pylori** ), and 1 yeast (Candida albicans) by the disc diffusion method. All of the synthesized compound exhibited different degrees of antimicrobial activity at concentrations between 20–100 μg/disc against the test organisms.

## 1. Introduction

**Figure 1:**
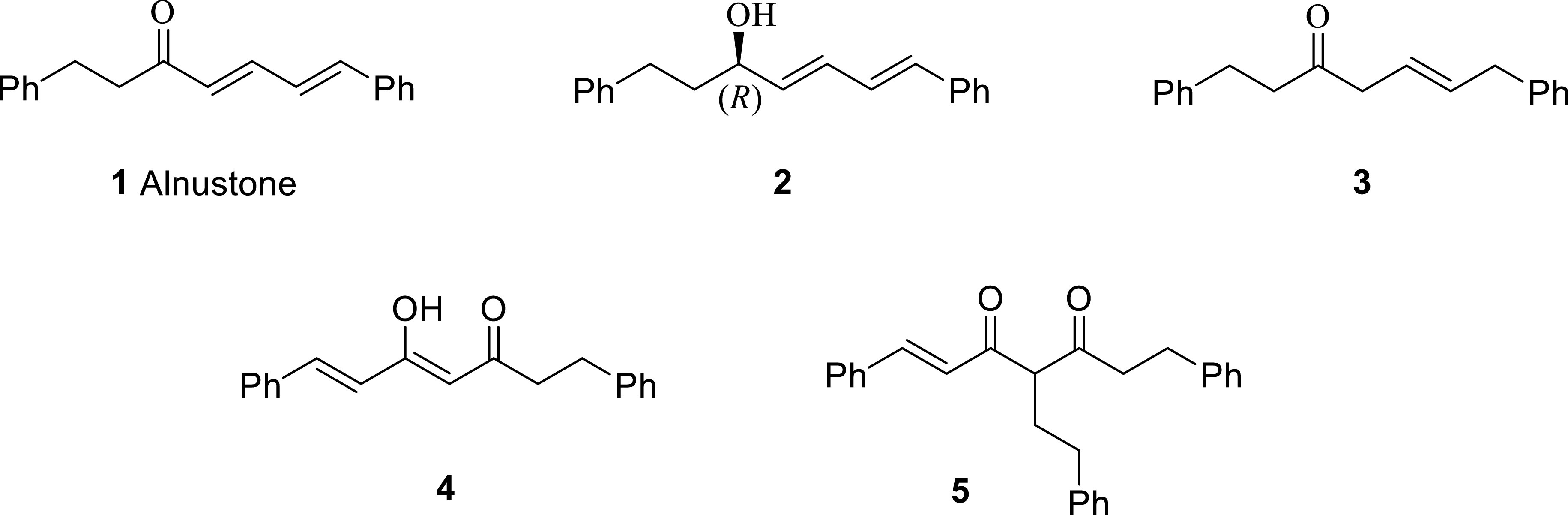
Alnustone-like natural nonphenolic diarylheptanoids.

Diarylheptanoids, a special class of natural products, are compounds with the structure of Ar-C_7_-Ar. Diarylheptanoids are classified into 4 categories: *i)* phenolic or nonphenolic linear diarylheptanoids, *ii)* macrocyclic diarylheptanoids, *iii)* macrocyclic diaryl ether heptanoids, and *iv)* C_7_ chain-cyclized diarylheptanoids [1]. Nonphenolic diarylheptanoids constitute a special group of linear diarylheptanoids. Alnustone ( **1** ) is one of the first examples of nonphenolic diarylheptanoids (Figure 1). The first isolation of alnustone was performed from *Alnus pendula* by Suga et al. [2,3]. Several studies showed that alnustone ( **1** ) is a bioactive molecule in a broad spectrum. On this basis, it has been reported to have antihepatotoxic [4], anti-inflammatory [5], antibacterial [6], antiemetic [7], and estrogenic activity [8].

(4 *E* ,6 *E* )-1,7-Diphenylhepta-4,6-dien-3-ol (-) ( **2** ), a natural diarylheptanoid, was first isolated from *Curcuma xanthorrhiza* by Claeson et al. [5] and was reported to exhibit significant antiinflammatory activity. Jurgens et al. [9] isolated compound **2** from *Curcuma comosa* . The following isolation of ( *R* )- **2** was performed by Suksamrarn et al. [8] and was reported to exhibit a good level of phytoestrogenic activity. In further studies, Tantikanlayaporn et al. [10,11] isolated ( *R* )- **2** from *Curcuma comosa* and reported its very strong phytoestrogenic activity.

Zhang et al. [12] reported ( *E* )-1,7-diphenyl-5-hepten-3-one ( **3** ) and compound 5 as new natural diarylheptanoids with strong antibacterial activity against *Helicobacter pylori* .

Compounds **4** and **5** are natural nonphenolic diarylheptanoids having a dihydrocurcumin structure from the aspect of the C-7 chain. Diarylheptanoid 4 was first isolated from the seeds of Alpinia katsumadai Hayata by Kuroyanagi et al. [13] and later isolated from Alnus maximowiczii [14], Alpinia officinarum Hance [15], and Curcuma ecalcarata [16]. Grienke et al. [17] reported ( *E,E* ) -5-hydroxy-1,7-diphenyl-4,6-heptadien-3-one, the ( *E,E* ) isomer of 4, to have strong neuraminidase inhibitory activity.

Alnustone ( **1** ) itself was synthesized from several starting materials using different synthetic methods [18–21]. In our previous studies we described an efficient method for the preparation of alnustone ( **1** ) itself [22] and alnustone-like compounds [23], and their antitumor activities against estrogen receptor alpha-positive human breast cancer.

Compound **4** was first synthesized by thermolysis of ethyl 3-oxo-5-phenyl-2-(cinnamoyl)pentanoate by Borsche and Lewinsohn [24]. Kato et al. [21] synthesized compounds **2** and **4** starting from 3-phenylpropanoic acid in many steps. A semisynthetic procedure for the preparation of ( *R/S* )- **2** was described by Yang et al. [25] via NaBH_4_ reduction of alnustone ( **1** ).

To the best of our knowledge, the enantioselective synthesis of R-( **2** ) and total synthesis of compound **3** are not known in the literature. In this study we report the first enantioselective synthesis of compound R-( **2** ), its enantiomer *S* -( **2** ), and **^3** via chemoselective reduction of alnustone ( **1** ). Moreover, we report an efficient one-pot synthesis of compound **4** and an attempted synthesis of compound **5** (Figure 1).

## 2. Results and discussion

**Figure 2:**
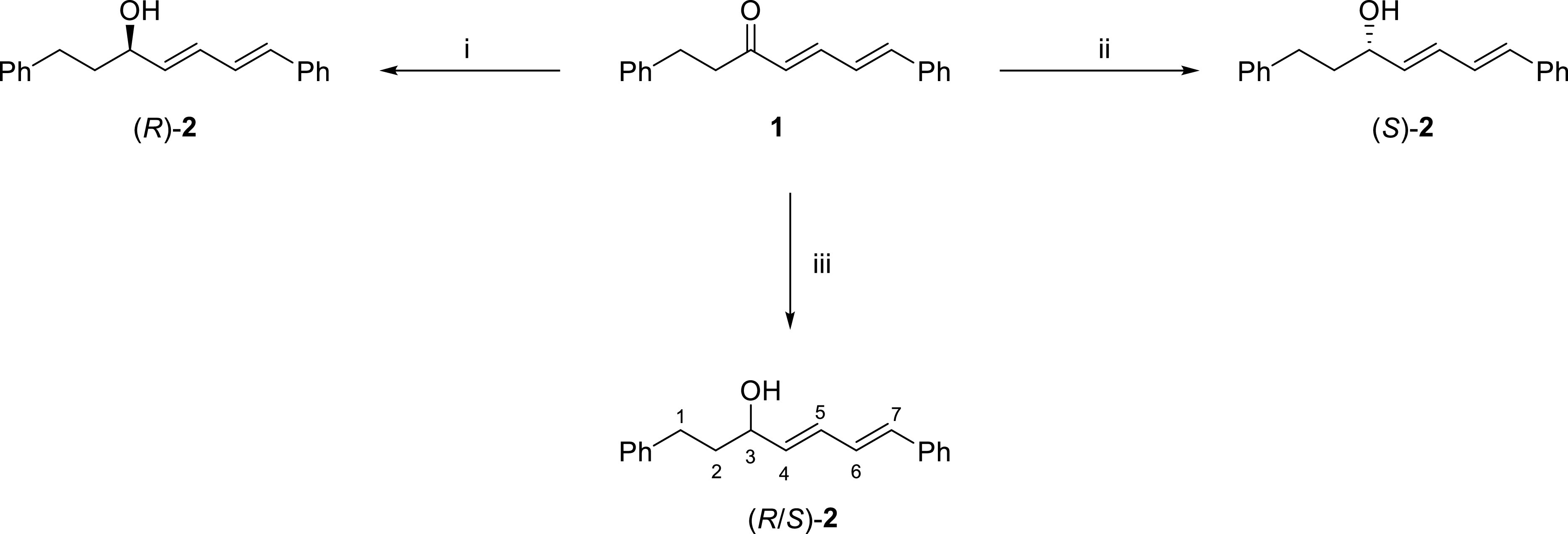
Syntheses of ( *R* )- **2** , ( *S* )- **2** , and ( *R/S* )- **2** . i) BH_3_.SMe_2_, ( *S* )-2-Me-CBS, THF, 0°C, N_2_ atm, 4 h, 80%; ii) BH_3_.SMe_2_, ( *R* )-2-Me-CBS, THF, 0°C, N_2_ atm, 4 h, 80%; iii) NaBH_4_, EtOH, 0°C, 4 h, 80%.

In our synthetic strategy, syntheses of compounds ( *R* )- **2** , ( *S* )- **2** , and **3** were based on reduction of alnustone ( **1** ). As mentioned above, efficient synthesis of alnustone ( **1** ) was previously reported by our research group [22,23]. If one considers the relationship between alnustone ( **1** ) and ( *R* )- **2** or ( *S* )- **2** , it can be readily seen that ( *R* )- **2** and ( *S* )- **2** could be obtained from enantioselective reduction of the carbonyl group of alnustone ( **1** ). Chiral oxazaborolidines have been used as reagents or catalysts (with borane sources) for enantioselective reduction of prochiral ketones [26]. In this manner, Corey et al. [27] efficiently used ( *S* )- **2** -methyl-CBS-oxazaborolidine as a catalyst with BH3 in the enantioselective reduction of aryl ketones to give the corresponding chiral alcohols with predominant R configuration (ee ~84-96.7%). We proposed that alnustone ( **1** ) itself may be successively reduced to give the corresponding alcohol ( *R* )- **2** by following this methodology. Indeed, while ( *S* )- **2** -Me-CBS-catalysed reduction of alnustone ( **1** ) gave alcohol ( *R* )- **2** (ee 90%), ( *R* )- **2** -Me-CBS-catalysed reduction gave alcohol ( *S* )- **2** (ee 90%) in good yield (80%) where the ee values were determined by chiral HPLC. The absolute configuration of alcohol ( *R* )- **2** was determined by agreement of its specific rotation with data given in the literature for natural ( *R* )- **2** [8]. The opposite specific rotation obtained for alcohol ( *S* )- **2** also confirmed its structure. The NaBH_4_ reduction [25] of alnustone ( **1** ) also gave racemic-2 in 80% yield (Figure 2).

For the structure of ( *R* )- **2** or ( *S* )- **2** , the ^1^H-NMR spectrum of the C_7_ chain showed 2 AB systems belonging to the olefinic HC(6)-HC(7) at δ 6.79 and δ 6.57 and to HC ( **4** )- HC ( **5** ) at δ 5.86 and δ 6.40, a quartet belonging to HC ( **3** ) at δ 4.24, and multiplets belonging to H_2_C( **1** ) at δ 2.81-2.68 and H_2_C( **2** ) at δ 2.05- 1.80. The absence of a carbonyl peak and the 3 aliphatic carbons at 72.2 (C( **3** )), 38.9 (C( **1** )), and 31.9 (C( **2** )) confirmed the reduction of the carbonyl group. Both ^1^H-NMR and ^13^C-NMR spectra of ( *S* )- **2** and ( *R* )- **2** were in agreement with the data given for ( *R* )- **2** in the literature [5,9]. Thus, we easily synthesized enantioselectively the natural compound ( *R* )- **2** and its enantiomer ( *S* )- **2** (Figure 2).

**Figure 3:**
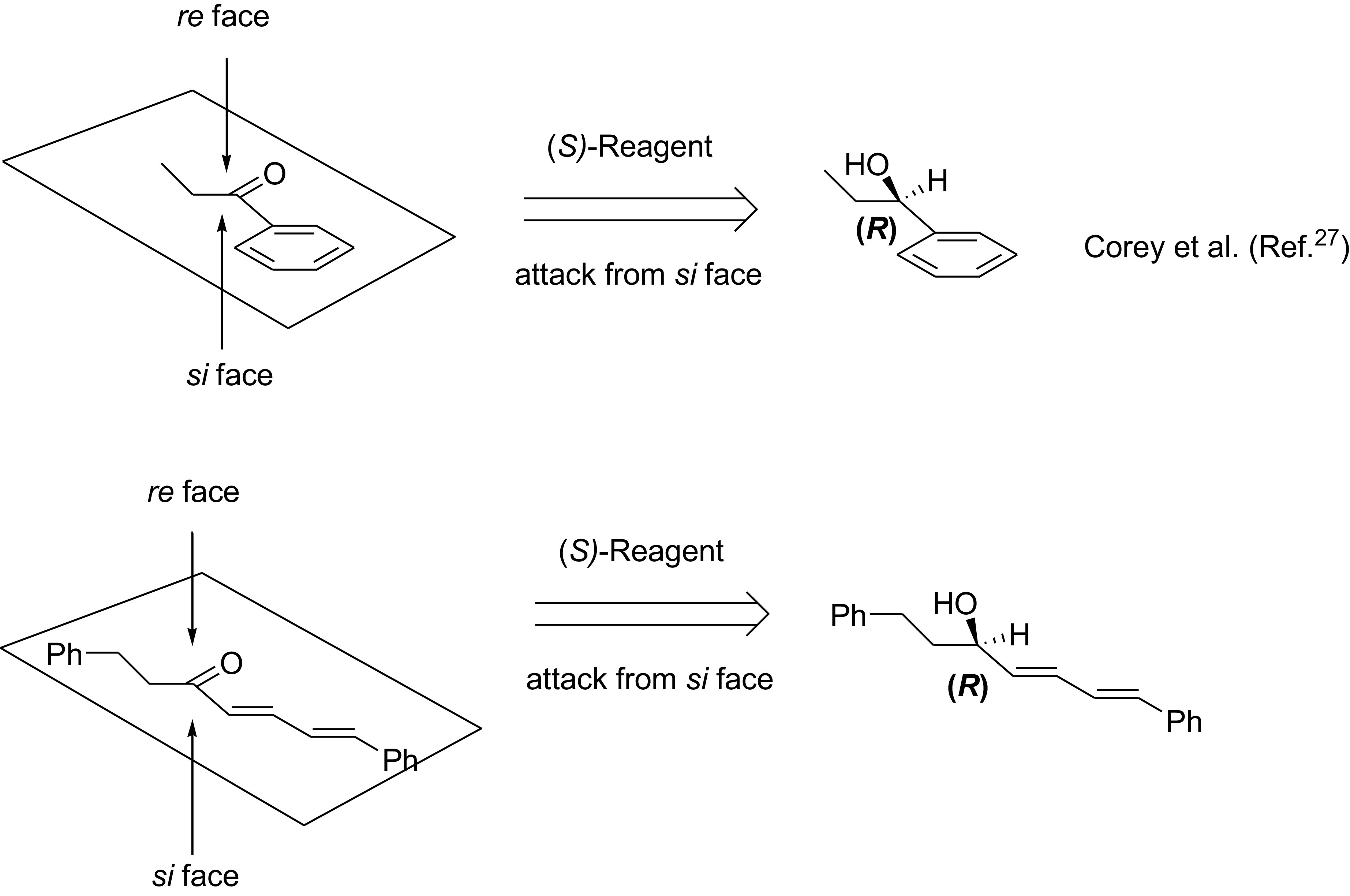
( *S* )-Oxazaborolidine-catalysed reduction of ethyl phenyl ketone and alnustone (1) with BH_3_.SMe_2_.

Mechanistically, the enantioselective formation of ( *R* )- **2** and ( *S* )- **2** is in good agreement with the results reported by Corey et al. [27]. As seen in Figure 3, both the ethyl phenyl ketone and alnustone ( **1** ) are reduced by hydride transformation from *si* faces.

**Figure 4:**
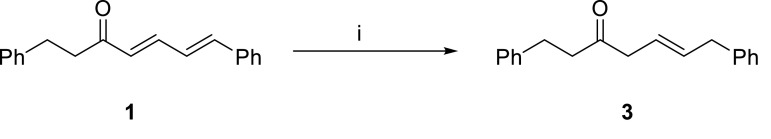
Preparation of compound **3** . i) Na, t-BuOH, dry ether, –15 °C, NH_3_ atm, 15 min, 85%.

We proposed that synthesis of compound **3** from alnustone ( **1** ) could be performed by a Birch reaction, which reduces 1,3-butadienes to 2-butenes. However, classical Birch reduction of alnustone ( **1** ) with metallic lithium in liquid N_3_ yielded a mixture with many undesirable reduction products. Therefore, instead of the classical Birch reduction conditions, the modification by Menzek et al. [28,29] was applied to obtain 3. This modification successfully allowed reduction to the conjugated 1,3-diene moiety of aromatic compounds using metallic lithium or sodium as the reducing agent in Et_2_O and *t* -BuOH under N_3_ (gas). Thus, reduction of alnustone ( **1** ) with 2.5 molar eq. Na as a reducing agent afforded compound **3** in a yield of 85% via Menzek’s method (Figure 4).

**Figure 5:**
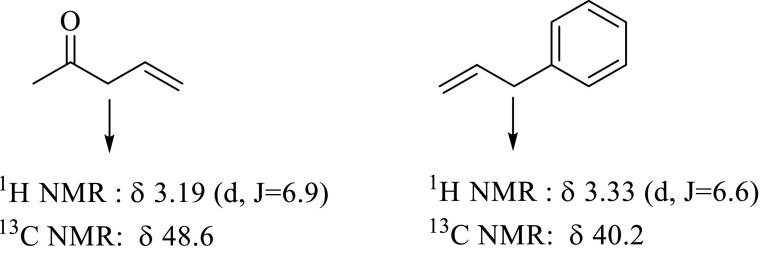
δ and J values of 4-penten-2-one and allylbenzene.

**Figure 6:**
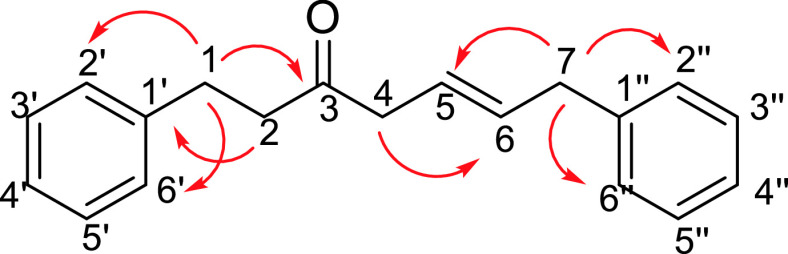
HMBC (H → C) correlations of compound 3.

The ^1^H-NMR spectrum of compound **3** was in exact agreement with its structure, in which olefinic hydrogens HC ( **5** ) at δ 5.67 and HC (6) at δ 5.74 arose as an AB system splitting into triplets (J_5,6_ = 15.4 Hz, J_4,5_ = 6.3 Hz, J_6,7_ = 6.2 Hz). In this context, H_2_C( **4** ) at δ 3.17 and H_2_C(7) at δ 3.43 also resonated as doublets. The spectral values belonging to H_2_C( **4** ) and H_2_C(7) of compound **3** are in good agreement with allylbenzene and 4-penten-2-one, 2 similar structures. In the structures, the CH_2_ of 4-penten-2-one resonates at δ 3.19 ppm with J = 6.9 Hz [30], and the CH_2_ of allylbenzene resonates at δ 3.33 ppm with J = 6.7 Hz [31] (Figure 5). In double-resonance experiments, irradiation of both olefinic protons together, HC( **5** ) and HC(6), converted doublets of H_2_C( **4** ) and H_2_C(7) to singlets, which supports the CH_2_-CH=CH-CH_2_ structure. H_2_C( **1** ) at δ 2.96 and H_2_C( **2** ) at δ 2.81 also resonated as triplets. The HR-ESI-MS spectrum of compound **3** was also in good agreement with the structure of compound **3** (C_19_H_20_NaO: 287.1418). HMBC correlations of compound **3** are given in Figure 6.

However, the ^1^H-NMR data of the heptenone part of compound **3** were in full disagreement with the reported data of Zhang et al. [12]. H/C assignments of the heptenone skeleton for synthetic compound **3** compared with data of the isolated 3 are given in Table 1. For example, they reported ^1^H-NMR data for H_2_C( **4** ) at δ 2.74 (d, J = 2.9 Hz, 2H) and H_2_C(7) at δ 2.77 (d, J = 2.9 Hz), which are also in disagreement with 4-penten-2-one and allylbenzene.

**Table 1:** H/C assignments of the heptenone skeleton for synthetic and isolated compound **3** .

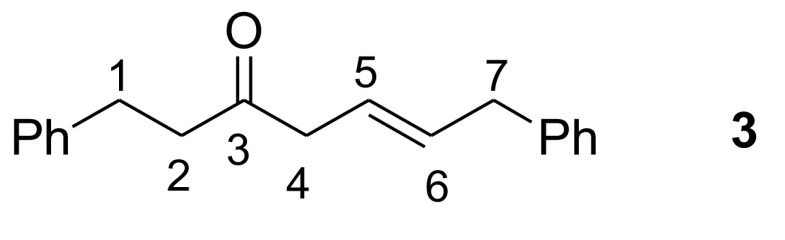		
H/C	Synthetic **3**	Isolated **3** (Ref.^12^)
1	2.96 (t, J = 7.5) 30.0	2.87–2.95 (m) 29.8 45.7
2	2.81 (t, J = 7.5) 44.0	
3	- 208.4	- 210.9
4	3.17 (d, J = 6.3) 47.1	2.74 (d, J = 2.9) 30.8
5	5.67 (dt, J = 15.4, 6.3) 123.6	6.10 (dt, J = 15.3, 7.0) 127.1
6	5.74 (dt, J = 15.4, 6.2) 134.0	6.12 (dt, J = 15.3, 7.0) 147.3
7	3.43 (d, J = 6.2) 39.3	2.77 (d, J = 2.9) 35.0

Nonetheless, for compound **3** isolated by Zhang et al. [12], the presence of 2 phenyl rings, 1 carbonyl, 4 CH_2_, 1 CH=CH, and especially the HRMS results are in agreement with C_19_H_20_O which implies a structural isomer of compound **3** .

Thus, we synthesized compound **3** starting from alnustone ( **1** ) in a 1-step reaction, which is the first reported synthetic method for compound **3** .

**Figure 7:**
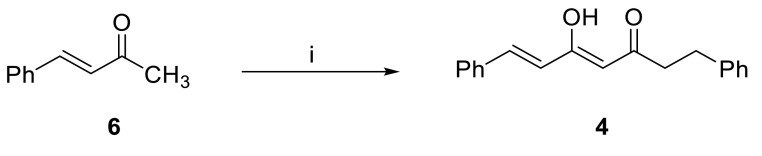
Synthesis of compound **4** . i) KOBu^t^, THF, 0 °C, 30 min; then 3-phenylpropanoyl chloride, rt, 12 h, 60%.

For the simple synthesis of compound **4** , we modelled a reaction described by Krishnamurty and Ghosh [32] for synthesis of dihydrocurcumin, in which they synthesized dihydrocurcumin via C-acylation of O-protected vanillylidene acetone with O-protected dihydroferuloyl chloride. We applied this methodology with a few modifications by treatment of benzalacetone (6) with KOBu^t^ at 0 °C and reacting with 3-phenylpropanoyl chloride to give compound **4** in a yield of 60% (Figure 7).

In the ^1^H-NMR spectrum of compound **4** the C_7_ chain has an AB system belonging to HC(7) at δ 7.60 and HC(6) at δ 6.46, a singlet belonging to HC( **4** ) at δ 5.64, and an A2 B2 system belonging to H_2_C( **2** ) at δ 2.74 and H_2_C( **1** ) at δ 3.00. The ^1^H-NMR and ^13^C-NMR spectra are in agreement with the data given for natural product 4 [13].

If we consider the similarity between compounds 4 and 5, we readily see that 5 is the alkylated derivative of compound **4** . Therefore, we proposed that we could readily obtain 5 by alkylating 4 with 2- phenylethylbromide. However, alkylation of 4 to give 5 with bases such as LDA, NaH, and KOBu^t^ was not successful. In another attempted synthesis, we proposed that an alkylation of 6 with PhCH_2_CH_2_ Br and then acylation with 3-phenylpropanoyl chloride could give compound 5. However, the first step of this attempted synthesis afforded styrene, an elimination product, instead of the expected ( *E* )-1,6-diphenylhex-1-en-3-one.

**Figure 8:**
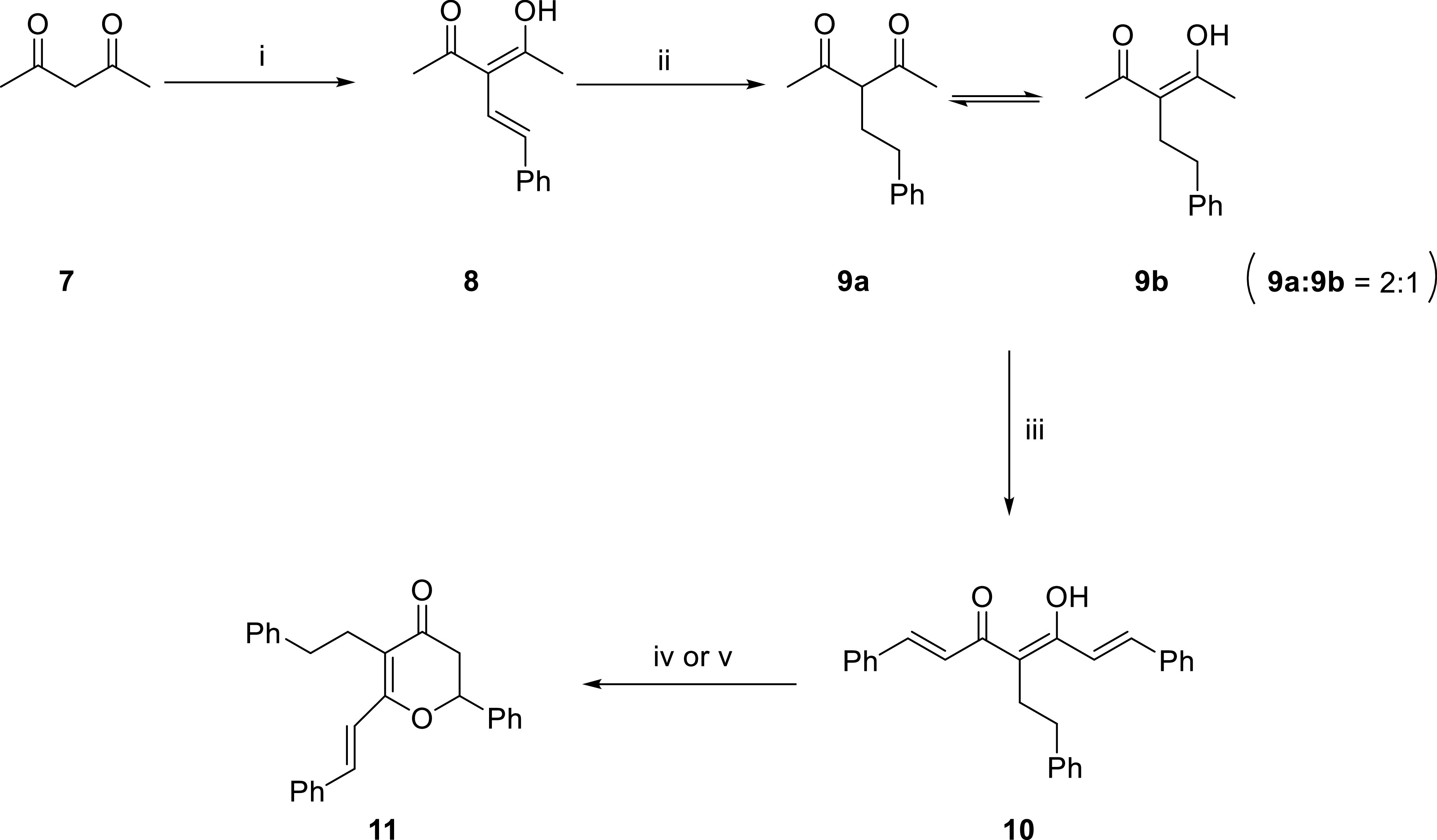
Attempted synthesis of compound **5** . i) 2-Phenylacetaldehyde, pyrrolidine, AcOH, benzene, 80 °C reflux, 18 h, 42%; ii) H_2_, Pd-C (cat), AcOEt, 25 °C, 3 h, 75%; iii) B_2_O_3_, benzaldehyde, morpholine, AcOH, microwave, 2450 MHz, 1 min, 34%; iv) Zn, AcOH, 118 °C reflux, 12 h, 76%; v) Zn(OAc)_2_ (1.1 molar eq.), AcOH, 118 °C reflux, 12 h, 70%.

Therefore, we decided to apply a different strategy, where 2,4-pentanedione was first condensed with 2- phenylacetaldehyde in the presence of AcOH and pyrrolidine to give 8 as a sole compound (Figure 8). Catalytic hydrogenation of 8 gave 9 as a keto-enol tautomer. Nichols et al. [33] developed a practical methodology for solvent-free microwave-assisted synthesis of curcumin analogues based on B_2_O_3_-mediated condensation of 2,4-pentanedione with benzaldehydes in the presence of morpholine and AcOH. Applying this methodology to 9 with benzaldehyde gave curcuminoid 10.

Curcuminoid 10 and natural product 5 differ from each other with a hydrogenated double bond. Therefore, we applied selective reduction of the double bonds of compound 10. Changtam et al. [34] reported an easy procedure for preparing dihydrocurcumin by reduction of curcumin with Zn in AcOH. However, applying this methodology to curcuminoid 10 gave cyclic compound 11. We proposed that this cyclization might occur with Zn(OAc)_2_ formed in situ in the reaction medium. Indeed, an independent reaction performed with Zn(OAc)_2_ supported this hypothesis with the formation of 11 from 10 in good yield (70%) (Figure 8). However, attempted selective reduction of compound 10 with Na or Mg to give 5, failed.

The synthesized chemical compounds 1, ( *R* )- **2** , ( *S* )- **2** , (R/S) -2, 3, 4, 10, and 11 were applied against 2 gram-positive bacteria ( *Bacillus cereus* and *Arthrobacter agilis* ), 4 gram-negative bacteria ( *Pseudomonas aeruginosa* , *Xanthomonas campestris* , *Klebsiella oxytoca* , and *Helicobacter pylori* ), and 1 yeast (Candida albicans) by disc diffusion method. The antimicrobial activity of the chemicals was variable, as seen in Table 2. All the synthesized chemicals showed different degrees of antimicrobial activity at concentrations of 30–90 μg/disc against the test organisms.

Each value is expressed as mean (n = 3). Inhibition zone was greater than 7 mm. These evaluations were carried out by diffusion disc tests.

**Table 2:** Minimum inhibitory concentrations (μg/disc) of tested compounds against selected microorganisms.

	
	1	*(S)* -1	*(R)* -2	*(R/S)* -2	3	4	10	11
Gram (-) bacteria	*H. pylori*	40	40	50	50	30	70	70	60
	*X. campestris*	60	60	60	50	50	70	70	60
	*K. oxytoca*	50	50	40	50	30	70	70	60
	*P. aeruginosa*	50	60	60	60	40	70	70	60
Gram (+) bacteria	*B. cereus*	30	40	40	30	30	80	70	70
	*A. agilis*	50	40	40	30	30	70	70	60
Yeast	*C. albicans*	40	60	60	50	40	90	70	70

Compound **3** was highly antibacterial against *K. oxytoca* , *A. agilis* , *B. cereus* , and *H. pylori* . Additionally, 3 and 1 were observed to have stronger inhibitory effects against C. albicans. Among the compounds, 4 was determined as the least effective against the microorganisms. Ofloxacin and nystatin are well known as antibacterial and antifungal substances, respectively. The average inhibition zones of ofloxacin and nystatin were found to be 22 and 20 mm, respectively.

In conclusion, we developed an easy synthesis of diarylheptanoids ( *R* )- **2** and ( *S* )- **2** , 3, and 4. An attempted synthesis of compound 5 gave a cyclocurcumin derivative (11).

The results of this study indicate that the synthesized chemicals showed dose-dependent inhibition against the tested microorganisms. This result may suggest that alnustone ( **1** ) and dihydroalnustone ( **3** ) structures can be developed for new antibiotic drugs.

## 3. Experimental

THF was used by distillation over Na. ^1^H- and ^13^C-NMR spectra were recorded with 400 (100) MHz Bruker and Varian instruments. Interchangeable hydrogens or carbons were shown with the same letters. Elemental analyses were performed with a LECO CHNS-932. HRMS spectra were recorded with an Agilent 6530 LC-MS QTOF. Enantiomeric excesses were determined by HPLC analysis using a chiral column eluting n-hexane–i-PrOH (90:10), and detection was performed at 210–254 nm. Optical rotations were measured on a Bellingham Stanley ADP220 589-nm spectropolarimeter. All percent yields were calculated from isolated compounds.

### 3.1. *(4E,6E)-1,7-Diphenylhepta-4,6-dien-3-one(alnustone)* (1)

Compound1was synthesized as described in our previous studies [22,23].

### 3.2. *(R,4E,6E)-1,7-Diphenylhepta-4,6-dien-3-ol (R)* -2

Under N_2_ atmosphere, 2 M BH_3_SMe_2_ in THF (0.12 mL, 0.24 mmol) was dissolved in THF (2 mL), cooled to 0 °C, and 1 M ( *S* )- **2** -Me-CBS in toluene (0.05 mL, 0.050 mmol) was added. After the reaction mixture was stirred at the same temperature for 1 h, alnustone ( **1** ) (100 mg, 0.38 mmol) was added to the reaction mixture. The resulting mixture was stirred for 4 h at 0 °C and MeOH (3 mL) was added, stirred for 15 min, and warmed to room temperature. The solvent was evaporated in vacuo, and a solution of saturated NH_4_Cl (2 mL) was added. The organic solution was extracted with AcOEt (3×25 mL) and dried (Na_2_SO_4_). The solvent was removed and the residue was purified by silica gel column chromatography with AcOEt/hexane (20:80) to give compound ( *R* )- **2** as a light-yellow oil (80 mg, 80%). Enantiomeric excess: 90%. R_f_ (AcOEt/hexanes 2:8): 0.4. [α]^27^_D_ = –53.3°(EtOH, c = 0.3 g/100 mL). Lit [8]: [α]^27^_D_ = –64.2°(EtOH, c = 0.3 g/100 mL). ^1^H-NMR (400 MHz, CDCl_3_) : δ 7.41–7.19 (m, 10 arom. H), 6.79 (dd, HC(6), J_6,7_ = 15.7, J_5,6_ = 10.6 Hz), 6.57 (d, HC(7), J_6,7_ = 15.7 Hz), 6.40 (dd, HC( **5** ), J_4,5_ = 15.2, J_5,6_ = 10.6 Hz), 5.86 (dd, HC( **4** ), J_4,5_ = 15.2, J3,4 = 7.0 Hz), 4.24 (q, HC( **3** ), J = 6.4 Hz), 2.81–2.68 (m, H_2_C( **1** )), 2.05–1.80 (m, H_2_C( **2** )) ppm; ^13^C-NMR (100 MHz, CDCl_3_) : δ 142.0 (C(1”)), 137.3 (C(1’)), 136.5 (C( **4** )), 133.0 (C(7)), 131.2 (C( **5** )), 128.8 (C(3’/5’)), 128.7 (C(2”/6”)), 128.6 (C(2’/6’)), 128.4 (C(4’)), 127.8 (C(4”)), 126.6 (C(3”/5”)), 126.1 (C(6)), 72.2 (C( **3** )), 38.9 (C( **1** )), 31.9 (C( **2** )) ppm; Anal. Calcd. for C_19_H_20_ O.1.3 H_2_O (MW 264.37): C, 79.3; H, 7.92%. Found: C, 78.94; H, 7.43%.

The ^1^H-NMR and ^13^C-NMR data are in agreement with data given in the literature [5,9].

### 3.3. *(R,4E,6E)-1,7-Diphenylhepta-4,6-dien-3-ol(S)-* 2

The procedure given above for synthesis of ( *S* )- **2** was applied using ( *R* )- **2** -Me-CBS to give compound ( *S* )- **2** (80 mg, 80%). R_f_ (AcOEt/hexanes 2:8): 0.4. Enantiomeric excess: 90%. [α]^27^_D_ = +53.3°(EtOH, c = 0.3 g/100 mL). ee: 0.90. Anal. Calcd. for C_19_H_20_ O.1.05 H_2_O (MW 264.37): C, 80.56; H, 7.86%. Found: C, 80.35; H, 7.63%.

### 3.4. *(4>E,6E)-1,7-Diphenylhepta-4,6-dien-3-ol(R/S)* -2

Alnustone ( **1** ) (300 mg, 1.14 mmol) was dissolved in EtOH and NaBH_4_ (50 mg, 1.31 mmol) was added, and the mixture was stirred for 4 h, monitored by TLC. The reaction mixture was cooled down to 0 °C and saturated aq. NH_4_Cl solution (2 mL) was added. The resulting aqueous solution was extracted with AcOEt (3×75 mL), and the organic phase was dried (Na_2_SO_4_). The solvent was removed and the residue was purified by silica gel column chromatography eluting with AcOEt/hexane (20:80) to give (R/S)-2 (0.250 g, 80%). R_f_ (AcOEt/hexanes 2:8): 0.5.

### 3.5. *(E)-1,7-Diphenylhept-5-en-3-one* (3)

Alnustone ( **1** ) (100 mg, 0.38 mmol) dissolved in dry Et_2_O (20 mL) was put into a 50-mL double-necked flask. A plastic balloon was connected to 1 neck of the flask and the other neck was fastened to a supply of N_3_ (gas). The solution was deoxygenated by the passing of 15 L of N_3_ (gas) and kept under N_3_ atmosphere by the filled balloon. The mixture was cooled to –15 °C, and then metallic Na (20 mg, 0.95 mmol) was added. At the same temperature, the reaction mixture was stirred for approximately 4 min, and then t-BuOH (70 mg, 0.95 mmol) was added and the reaction mixture was stirred for 16 min. At this stage, the colour of the reaction mixture changed to dark green as monitored by TLC and the completion of the reaction was observed. EtOH (15 mL) was added to quench the reaction mixture and then all solvents were removed under reduced pressure. The residue was dissolved in H_2_O (5 mL) and extracted with AcOEt (4 ×15 mL). The combined organic phase was dried on Na_2_SO_4_ and filtered. Evaporation of the solvent gave a crude product, which was then chromatographed on a silica gel column eluting with 95:5 AcOEt/hexane solvent mixture to give light yellow oily compound **3** (85 mg, 85%). R_f_ (AcOEt/hexanes 1:9): 0.64. ^1^H-NMR (400 MHz, CDCl_3_) : δ 7.37–7.23 (m, 10 arom. H), 5.74 (dt, A of AB, HC(6), J_5,6_ = 15.4 Hz, J_6,7_ = 6.2 Hz), 5.67 (dt, B of AB, HC( **5** ), J_5,6_ = 15.4 Hz, J_4,5_ = 6.3 Hz), 3.43 (d, H_2_C(7), J_6,7_ = 6.2 Hz), 3.17 (d, H_2_C( **4** ), J_4,5_ = 6.3 Hz), 2.96 (t, H_2_C( **1** ), J = 7.5 Hz), 2.81 (t, 2H, H_2_C( **2** ), J = 7.5 Hz) ppm; ^13^C-NMR (100 MHz, CDCl_3_) : δ 208.4 (C( **3** )), 141.3 (C(1’)), 140.4 (C(1”)), 134.0 (C(6)), 128.80 (C(3’/5’)), 128.78 (C(3”/5”)), 128.6 (C(2’/6’)), 128.4 (C(2”/6”)), 126.4 (C(4’/4”)), 123.6 (C( **5** )), 47.1 (C( **4** )), 44.0 (C( **2** )), 39.3 (C(7)), 30.0 (C( **1** )) ppm; Anal. Calcd. for C_19_H_20_ O (MW 264.37): C, 86.32; H, 7.63%. Found: C, 86.96; H, 7.80%. HR-ESI-MS: 287.1418 [M+Na]+; calc. 287.1406.

### 3.6. *(4Z,6E)-5-Hydroxy-1,7-diphenylhepta-4,6-dien-3-one* (4)

Under N_2_ atmosphere, a solution of KOBu^t^ (300 mg, 2.65 mmol) in anhydrous THF (15 mL) was cooled to 0 °C and ( *E* )-4-phenylbut-3-en-2-one (6) (200 mg, 1.37 mmol) in anhydrous THF (20 mL) was added to the solution. The mixture was stirred for 30 min, and then 3-phenylpropanoyl chloride (230 mg, 1.37 mmol) was added. The mixture was stirred at room temperature for 12 h. An aq. sat. NH_4_Cl solution (3 mL) was added to the mixture at 0 °C. THF was evaporated, and the organic phase was extracted with AcOEt (2 ×50 mL) and dried over Na_2_SO_4_ . Evaporation of the solvent and silica gel column chromatography of the residue with hexane/AcOEt 95:5 gave 4 as light-yellow crystals (228 mg, 60%). R_f_ (AcOEt/hexanes 1:9): 0.80. Mp 67–68 °C (Lit13 : 74–75 °C from hexane). 4: ^1^H-NMR (400 MHz, CDCl_3_) : δ 15.3 (s, 1H, OH), 7.60 (d, 1H, HC(7), J = 15.7 Hz), 7.42–7.20 (m, 10 arom. H), 6.46 (d, 1H, HC(6), J = 15.7 Hz), 5.64 (s, 1H, HC( **4** )), 3.00 (t, 2H, H_2_C( **1** ), J = 7.8 Hz), 2.74 (t, 2H, H_2_C( **2** ), J = 7.8 Hz) ppm; ^13^C-NMR (100 MHz, CDCl_3_) : δ 199.9 (C( **3** )), 176.7 (C( **5** )), 140.8 (C(1”)), 139.8 (C(7)), 135.1 (C(1’)), 129.9 (C(4’)), 128.9 (C(3’/5’a)) , 128.5 (C(2’/6’a)) , 128.3 (C(3”/5”a)) , 127.9 (C(2”/6”a)) , 126.2 (C(4”)), 122.7 (C(6)), 100.8 (C( **4** )), 41.9 (C( **2** )), 31.2 (C( **1** )) ppm. The ^1^H-NMR and ^13^C-NMR data are in agreement with the data given for isolated natural product 4 [13]. Anal. Calcd. for C19 H18 O2 (MW 278.35): C, 81.99; H, 6.52%. Found: C, 82.13; H: 6.53%.

### 3.7. *(Z)-4-Hydroxy-3-((E)-styryl)pent-3-en-2-one* (8)

To pentan-2,4-dione (7) (7.000 g, 69.9 mmol) in benzene (50 mL) was added 2-phenylacetaldehyde (8.40 g, 69.9 mmol), a catalytic amount of anhydrous AcOH (0.35 mL), and pyrrolidine (0.56 mL). Using a Dean-Stark apparatus the reaction mixture was refluxed for 18 h with monitoring by TLC. After cooling to ambient temperature, the mixture was diluted with Et_2_O (50 mL) and H_2_O (25 mL). The organic layer was separated and washed with H_2_O (25 mL), 1 M HCl (2 ×25 mL), and a saturated solution of NaHCO_3_ (10 mL). The organic layer was dried over Na_2_SO_4_ . Evaporation of the solvent and column chromatography of the residue with hexane/AcOEt 9:5 gave 8 as a dark yellow oil (5.96 g, 42%). R_f_ (AcOEt/hexanes 1:9): 0.60. ^1^H-NMR (400 MHz, CDCl_3_) : δ 16.74 (s, 1H, OH), 7.44–7.34 (m, 5 arom. H), 6.75 (d, 1H, styryl, J = 16.1 Hz), 6.42 (d, 1H, styryl, J = 16.1 Hz), 2.22 (s, 6H, 2 ×CH_3_) ppm; ^13^C-NMR (100 MHz, CDCl_3_) : δ 191.4 (C(2/4)), 137.4 (C(1”, Ph)), 134.5 styryl (C( **1** )), 128.9 (C(3”/5”, Ph)), 128.0 (C(2”/6”, Ph)), 126.4 (C(4”, Ph)), 123.1 styryl (C( **2** )), 111.6 (C( **3** )), 24.5 (C(1/5)) ppm. The ^1^H-NMR and ^13^C-NMR data are in agreement with data given in the literature [35].

### 3.8. *(Z)-4-Hydroxy-3-phenethylpent-3-en-2-one* (9)

Pd/C (10%) (69 mg) was added to EtOAc (50 mL) cooled to 0 °C and then (Z)-4-hydroxy-3-(( *E* )-styryl)pent-3-en-2-one ( **8** ) (690 mg, 3.41 mmol) in AcOEt (20 mL) was added to the 100-mL double-necked flask. A balloon filled with H2 gas (3 L) was fitted to the flask. The mixture was deoxygenated by flushing with H2 and then hydrogenated for 3 h at room temperature. When TLC analyses indicated the disappearance of the starting material, the catalyst was removed by filtration. Removal of the solvent under reduced pressure gave 9 as a keto-enol tautomer and light-yellow oil (( **9a** ):( **9b** ) = 2:1) (520 mg, 75%). 9a: ^1^H-NMR (400 MHz, CDCl_3_) : δ 7.31–7.15 (m, 5 arom. H), 3.63 (t, 1H, HC( **3** ), J = 7.3 Hz), 2.70 (t, 2H, Ph CH_2_CH_2_, J = 7.8 Hz), 2.22–2.12 (CH CH_2_CH_2_ Ph), 2.14 (s, 6H, 2×CH_3_) ppm; ^13^C-NMR (100 MHz, CDCl_3_) : δ 204.3 (C(2/4)), 141.2 (C(1” of Ph)), 128.8 (C(3”/5” of Ph)), 128.8 (C(2”/6” of Ph)), 126.6 (C(4” of Ph)), 68.1 (C( **3** )); 33.7 (Ph CH_2_CH_2_), 29.9 (PhCH_2_ CH_2_), 29.4 (C(1/5)) ppm. **9b** : ^1^H-NMR (400 MHz, CDCl_3_) : δ 16.83 (s, 1H, OH), 7.31-7.15 (m, 5 arom. H), 2.59–2.52 (A2 B2 system, 4H, CH_2_CH_2_) , 2.03 (s, 6H, 2 ×CH_3_) ppm; ^13^C-NMR (100 MHz, CDCl_3_) : δ 191.6 (C(2/4)), 140.8 (C(1” of Ph)), 128.8 (C(3”/5” of Ph)), 128.7 (C(2”/6” of Ph)), 126.5 (C(4” of Ph)), 109.6 (C( **3** )), 37.0 (Ph CH_2_CH_2_), 29.9 (PhCH_2_ CH_2_) , 22.9 (C(1/5)), 2.81 (t, 2H, H_2_C( **2** ), J = 7.5 Hz), 5.74 (dt, A of AB, HC(6), J_5,6_ = 15.4 Hz, J_6,7_ = 6.2 Hz).

### 3.9. *(1E,4Z,6E)-5-Hydroxy-4-phenethyl-1,7-diphenylhepta-1,4,6-trien-3-one* (10)

Compound **9** (1.2 g, 5.87 mmol) was mixed with boric anhydride (400 mg, 5.74 mmol) in a 250-mL Erlenmeyer flask. Benzaldehyde (1.25 g, 1.2 mL, 11.77 mmol), morpholine (300 mg, 0.3 mL, 3.44 mmol), and acetic acid (210 mg, 0.2 mL, 3.5 mmol) were then added to the mixture. The reaction was irradiated in a kitchen-type microwave (2450 MHz) for 1 min. The flask was cooled and then MeOH (30 mL) was added. This mixture was then sonicated in a sonicator for 10 min. The suspended mixture was taken onto filter paper and washed with MeOH cooled to 0 °C, and then the MeOH-soluble part was removed. The solid part remaining on the paper was dried in air. Thus, compound **10** was obtained as a sole enol form (0.75 g, 34%). Yellow crystal. Mp 151–152 °C. ^1^H-NMR (400 MHz, CDCl_3_) : δ 17.60 (s, 1H, OH), 7.73 (d, 2H, HC(1/7), J = 15.4 Hz), 7.53–7.17 (m, 15 arom. H), 6.98 (d, 2H, HC(2/6), J = 15.4 Hz), 2.88 (A2 B2 , quasi s, 4H, CH_2_CH_2_) ppm; ^13^C-NMR (100 MHz, CDCl_3_) : δ 183.4 (C(3/5)), 141.8 (C(1/7)), 141.0 (C(1”’)), 135.6 (C(1’/1”)), 130.2 (C(2”’/6”’)), 129.1 (C(2’/6’ and 2”/6”)), 128.9 (C(3”’/5”’)), 128.8 (C(4’/4”)), 128.4 (C(3’/5’ and C-3”/5”)), 126.6 (C(4”’)), 120.6 (C(2/6)), 110.9 (C( **4** )), 38.6 (Ph CH_2_CH_2_), 28.4 (PhCH_2_ CH_2_) ppm. Anal. Calcd. for C_27_H_24_O_2_ (MW 380.49): C, 85.23; H, 6.36%. Found: C, 84.96; H, 6.60%.

### 3.10. *(E)-5-Phenethyl-2-phenyl-6-styryl-2,3-dihydro-4H-pyran-4-one* (11)

Compound 10 (50 mg, 0.13 mmol) was dissolved in AcOH (10 mL). Zn (10 mg, 0.15 mmol) was added to this solution, and the reaction mixture was refluxed for 12 h. After the mixture was cooled to room temperature, H_2_O (25 mL) was added. The organic phase was extracted with AcOEt (2 ×25 mL) and washed with H_2_O (20 mL). The organic layer was dried (Na_2_SO_4_), and evaporation of the solvent and silica gel column chromatography of the residue with hexane/AcOEt 95:5 gave 11 as a colourless oil (38 mg, 76%). R_f_ (AcOEt/hexanes 5:95): 0.6. ^1^H-NMR (400 MHz, CDCl_3_) : δ 7.52–7.16 (m, 15 arom. H and 1H, styryl), 6.75 (d, 1H, styryl, J = 15.8 Hz), 5.41 (dd, 1H, HC( **2** ), J = 13.9, J = 3.3 Hz), 2.94 (dd, 1H, HC(3(a)), J = 17.0, J = 13.9 Hz), 2.81–2.72 (m, 5H, CH_2_CH_2_ and HC(3(b)); ^13^C-NMR (100 MHz, CDCl_3_) : δ 192.6 (C( **4** )), 164.6 (C(6)); 142.0 (C(1”)), 139.2 (C(1’)), 137.4 (Ph CH =CH), 136.0 (C(1”’)), 129.63 (1 arom. C), 129.04 (4 arom. C), 128.95 (2 arom. C), 128.88 (1 arom. C), 128.62 (2 arom. C), 127.9 (2 arom. C), 126.3 (2 arom. C), 126.29 (1 arom. C), 119.0 (PhCH= CH ), 116.2 (C( **5** )), 79.6 (C( **2** )), 43.7 (C( **3** )), 36.4 (Ph CH_2_CH_2_) , 26.2 (PhCH_2_ CH_2_) ppm; Anal. Calcd. for C_27_H_24_O_2_ (MW 380.49): C, 85.23; H, 6.36%. Found: C, 85.46; H, 6.53%.

### 3.11. Antimicrobial activity

The synthesized chemicals were dissolved in dimethyl sulfoxide (DMSO, 80%) at a final concentration of 10 mg/mL. Antimicrobial activities of the synthesized chemicals were tested by disc diffusion method against *Bacillus cereus* BC-On [36], *Arthrobacter agilis* A17 [37], *Pseudomonas aeruginosa* OG1 [38], *Xanthomonas campestris* MO-03 [39], Candida albicans, *Klebsiella oxytoca* (clinical isolates), and *Helicobacter pylori* [40] (ATCC 43629). For this purpose, a suspension of the tested microorganism (0.1 mL of 10^8^ cells per mL) was spread on the agar plates. Mueller-Hinton agar with 5% sheep blood plates for *H. pylori* and tryptic soy agar (TSA) for the other bacteria were used for testing antibacterial activities. *H. pylori* plates were incubated under microaerophilic conditions in anaerobic jars [40]. Discs made of Whatman qualitative filter paper, grade 1 (6 mm diameter), containing 20–100 μg/disc of the synthesized chemicals were placed on the plates. The plates were incubated at 37 °C for 24 h. At the end of the incubation period, a zone of inhibition of diameter ≥7 mm was considered positive. DMSO was used as the negative control. Ofloxacin and nystatin (HiMedia, India) were used as positive standards for antimicrobial activities.
